# FedHGCDroid: An Adaptive Multi-Dimensional Federated Learning for Privacy-Preserving Android Malware Classification

**DOI:** 10.3390/e24070919

**Published:** 2022-07-01

**Authors:** Changnan Jiang, Kanglong Yin, Chunhe Xia, Weidong Huang

**Affiliations:** 1 Key Laboratory of Beijing Network Technology, Beihang University, Beijing 100191, China; jcnby@buaa.edu.cn (C.J.); yinkanglong@buaa.edu.cn (K.Y.); bigeast@buaa.edu.cn (W.H.); 2 Guangxi Key Lab of Multi-Source Information Mining and Security, Guangxi Normal University, Guilin 541004, China

**Keywords:** federated learning, malware classification, call graph, adaptive

## Abstract

With the popularity of Android and its open source, the Android platform has become an attractive target for hackers, and the detection and classification of malware has become a research hotspot. Existing malware classification methods rely on complex manual operation or large-volume high-quality training data. However, malware data collected by security providers contains user privacy information, such as user identity and behavior habit information. The increasing concern for user privacy poses a challenge to the current malware classification scheme. Based on this problem, we propose a new android malware classification scheme based on Federated learning, named FedHGCDroid, which classifies malware on Android clients in a privacy-protected manner. Firstly, we use a convolutional neural network and graph neural network to design a novel multi-dimensional malware classification model HGCDroid, which can effectively extract malicious behavior features to classify the malware accurately. Secondly, we introduce an FL framework to enable distributed Android clients to collaboratively train a comprehensive Android malware classification model in a privacy-preserving way. Finally, to adapt to the non-IID distribution of malware on Android clients, we propose a contribution degree-based adaptive classifier training mechanism FedAdapt to improve the adaptability of the malware classifier based on Federated learning. Comprehensive experimental studies on the Androzoo dataset (under different non-IID data settings) show that the FedHGCDroid achieves more adaptability and higher accuracy than the other state-of-the-art methods.

## 1. Introduction

### 1.1. Background and Motivation

Android dominated the mobile phone market in 2021 with an 84 percent market share. Due to high usage and an open-source development ecosystem, it has become an effective way for hackers to create malware that accesses sensitive user information, such as geographical position and contact information. Unlike other closed-source platforms such as IOS, hackers can examine an app’s source code and exploit vulnerabilities to develop malware [1]. In addition, users may download unknown third-party apps on Android devices, causing Android malware to spread more widely. Therefore, mobile anti-malware methods are crucial to Android users.

Conventional anti-malware methods mainly use signature-based classification technology [2], which relies on analyzing attack signatures extracted by experts. However, conventional classification methods fail to mitigate newly generated malware, such as zero-day malware. In contrast, machine-learning-based anti-malware methods using dynamic features and static features (such as API calls, and permissions) can effectively identify unknown malware. In recent years, security experts have introduced deep-learning-based methods to achieve accurate malware classification. Compared with the previous static and dynamic conventional machine learning models, deep-learning-based methods can automatically extract critical features without expert domain knowledge [3], so they have gradually become a hotspot for anti-malware solutions in recent years.

However, due to the ambiguity of malicious behavior and the evolution of malware over time (such as the change of API version used), the existing classification methods of malware still have the problem of insufficient mining of malicious behavior characteristics, which leads to low classification performance.

Existing schemes, on the other hand, rely on a large amount of artificial signature or the vast amount of high-quality data available. However, Android users are reluctant to share their compromised malware data due to privacy concerns, a strict Privacy Protection Act has been put in place, so data exist as islands. The limitation of the data sources used for training severely reduces the performance of the malware classifier. In addition, people tend to ignore sensitive private information in training data, leading to potential privacy leakage [4,5].

Federated learning [6,7] is a novel approach to solving the privacy problems of existing malware classification models, which does not require access to users’ private data. However, the differences in users’ (from the different organizations or communities) identities or preferences will lead to heterogeneity distribution (non-IID) between the local malware type distribution. Existing FL-based schemes have poor adaptability to such non-IID scenarios, and the classification accuracy will be greatly reduced.

In order to solve the above problems, this work proposes an adaptive multi-dimensional FL-based malware classification framework, FedHGCDroid. Unlike the existing centralized malware classification framework, FedHGCDroid uses the Federated learning framework to train the malware classifier without accessing the user’s private data, which solves the problem of user privacy disclosure in the existing scheme. At the same time, FedHGCDroid innovatively combined the statistical features and graph features of malware and carried out multi-attribute coding on the API to mine the malicious behaviors of malware more effectively. It solved the problem that the existing framework was not comprehensive and effective enough to mine the behaviors of malware, thus improving the overall classification accuracy. Finally, FedHGCDroid uses an adaptive contribution degree-based classification model training mechanism (FedAdapt), which innovatively improves the existing Fedavg algorithm by introducing meta-learning and attention mechanisms. It solved the problem of poor adaptability of the existing FL-based framework to non-IID distribution. 

The rest of this article is organized as follows. Section 1 provides an overview of malware classification and Federated learning. In Section 2, we introduce the system model and describe corresponding limitations. In Section 3, we introduce the proposed adaptive FL-based android malware classification method. In Section 4, we describe our experimental setup and results. Finally, in Section 5, we present our conclusions and future work.

### 1.2. Related Work

In this section, we first review the latest work in the field of machine learning (centralized) methods for malware classification and detection, as a reference source and inspiration for our framework. Secondly, the latest work of malware classification schemes based on Federated learning is reviewed, and the limitations and improvement direction of existing Federated learning schemes under the task of malware classification are analyzed. 

#### 1.2.1. Detection and Classification Method of Android Malware Based on ML

Existing Android malware detection methods fall into two categories, either static analysis, with no code execution, or dynamic analysis, with an app executed in real-time and its behavior studied [8]. ML models were adopted in early work, including SVM, DNN, Rotation Forest, LSTM, and GNN.

Dynamic features can reflect the runtime behavior of applications. Cai et al. [9] presented a novel classification approach (DroidCat) which is based on dynamic analysis. The authors used a set of dynamic features such as method calls, app resources, and Inter-Component Communication. In [10], the authors proposed a dynamic analysis framework EnDroid, which used different types of dynamic features for the identification of malware. They employed a chi-square algorithm to select the relevant features and applied an ensemble learning technique to differentiate between malware and benign apps. However, these technologies require running a sandbox for behavior detection, which can affect a host running in real-time. At the same time, dynamic analysis cannot cover all program’s behavior.

In contrast, the method based on static features does not need to run the app for behavior checking. Static features can be obtained simply by analyzing an Android Application Pack (APK). Opcode [11], permission requirements, intent actions, and APIs, as the most common low-level static features, are widely used in Android malware detection. Arp [12] proposed DREBIN, which uses Android permissions and sensitive APIs for extensive static analysis. DREBIN is a lightweight method that detects malware on smartphones. In [13], Android malware is classified using n-gram features of operation sequences. However, this approach is not robust against obfuscation because the opcode sequence can be modified easily. In [14], the authors suggested a highly efficient method to extract API calls, permission rate, surveillance system events, and permissions as features. They constructed a model based on ensemble Rotation Forest to identify whether an app is malicious or benign.

However, the API is simply represented as a binary vector in which each element indicates whether the appropriate API is invoked or not. Although API-based vector representations can reveal some information about an application, they cannot describe the interactions between functions, so they cannot tell the application’s behavior effectively, and the robustness of hiding and evading operations for malware authors is poor, resulting in poor classification results.

In contrast, high-level structural features such as behavior diagrams are considered more robust because they are relatively expensive to modify. Behavior diagrams provide topology information that can be used to infer the runtime behavior of an application. In the method of behavior-graph-based malware classification, researchers mainly detect malware through various behavior pattern diagrams corresponding to software.

Nguyen [15] extracted the control flow diagram from the binary code and obtained the corresponding adjacency matrix, and then extracted three specified features from the control flow diagram as RGB channel features so as to transform the control flow diagram into RGB images, which were finally input to the convolutional neural network for malware detection. However, the method relies on professional domain knowledge for algorithm design, and the classification accuracy rate also fluctuates sharply with the accuracy of feature selection and the quality of the graph matching algorithm.

With the popularity of graph representation learning [16,17], researchers have begun to pay attention to using graph models to learn behavior graph features automatically. Jiang [18] used the double-stacked denoising autoencoder to obtain the embedded representation of a function call graph, spliced the feature vector of the function call, and then input it into the deep neural network for malware classification. Pektas [19] used an API call graph as a graphical representation of all possible execution paths that the malware could track during its run. The API call graph is embedded into the deep neural network and transformed into a feature set of low-dimensional numerical vectors.

The above classification method based on behavior graph relies on a single type of feature that can only capture one aspect of the application behavior, which hackers can easily escape. This will lead to the failure of the local model to achieve the desired classification accuracy and further affect the convergence efficiency and classification accuracy of the global model in federated learning.

Based on the above literature analysis, it is still difficult for existing malware classification schemes to comprehensively and effectively mine the behavior of malware, which will restrict the classification effect of machine learning schemes and the application efficiency in the Federated learning framework. A summary including previous related review articles on detecting malware is provided in Table 1 and Table 2.

#### 1.2.2. Malware Classification Method Based on Federated Learning

Existing methods that use ML/DL to classify malware rely on the vast amount of high-quality available data from different clients to train the accurate global model. These models are then distributed to individual clients, or these clients upload their test data to the server for real-time behavior checking and malware classification. However, training data contains private information about user behavior, which will seriously affect user security and privacy once it falls into the hands of malicious elements.

To solve users’ privacy concerns in deep learning, Google [24] proposed Federated learning, a collaborative learning approach that ensures privacy by storing it locally on the client. In the Federated learning (FL) approach, each client executes local training using its local ML classifier model to generate local weights. The client uploads these local weights to the FL server. The FL server runs an average calculation on these local features and returns a usable global model. In FL, sharing local weights rather than raw data ensures users’ privacy. 

Taheri [23] proposed a robust FL-based framework, namely, Fed-IIoT, for detecting Android malware in the Internet of Things. Fed-IIoT forms a robust cooperative training model by adjusting two GAN-based adversarial algorithms. Narendra [20] proposes a lightweight model based on a convolutional neural network (CNN), which uses a call graph, N-gram, and image transformation to extract relevant features. In addition, the author designed an auxiliary classifier generative adversarial network (AC-GAN) to generate invisible data for training. Shukla [22] introduces a performance-aware FL framework to reduce the communication overhead of device-level computing. Singh [25] uses the FL framework to train a web security model from users’ browsing data and share it with a centralized server. Valerian [26] proposed a privacy-preserving framework for malware detection in the Internet of Things, and their aggregation function based on mean pruning is tested as a countermeasure against adversarial attacks.

Galvez [21] presented LiM, the federated learning algorithm that works without user supervision, making use of safe semi-supervised learning techniques.

However, most of the studies mentioned above use FL as a base model, and they have ignored the problem of adaptation to the non-IID distribution of malware on different clients. Although some schemes were regularized or pruned for outliers, their classification models did not adapt to the local specific distribution. As a result, it will lead to a long convergence time for the FL-based malware classification model [27], and the classification accuracy of different clients is not ideal. Considering the complexity of real situations, FL-based malware classification schemes need to deal with various data distribution scenarios. Therefore, it is necessary to develop an FL framework that is adaptive to the non-IID distribution of malware on Android clients, to achieve the accurate and adaptive classification of malware.

### 1.3. Contribution

The contributions of this paper are summarized below.

(1) We introduce an FL framework to develop an Android malware collaborative classification model, which realizes proper privacy preservation of data resources according to GDPR principles. 

(2) We propose a novel multi-dimensional malware classification model, namely HGCdroid (as a local classification model in the proposed FL framework). The model uses the CNN network to capture the statistical features of malware and the GNN network to capture the graphical features of malware to obtain effective behavioral features of Android malware and then classify it accurately. HGCDroid can achieve up to 91.3% accuracy and 91.25% F1-score in malware detection tasks (on the Androzoo dataset), higher than the baseline model. To the best of our knowledge, this is the first attempt to combine statistical features with multi-attribute graph features for malware classification.

(3) We innovatively combine the idea of meta-learning and attentional mechanisms and propose a contribution degree-based adaptive classifier training mechanism, namely FedAdapt. The FedAdapt improves the adaptive performance of the proposed framework in scenarios with non-IID distributions on different clients. To the best of our knowledge, this is the first attempt to combine attentional mechanisms with meta-learning for adaptive performance optimization of the FL-based malware classification framework.

(4) We analyze the scenarios of non-IID distributions in the malware classification field and propose three dataset partitioning strategies for non-IID distributions scenarios; we also propose a scheme to measure the degree of non-IID distributions. Finally, we conducted extensive experiments (on the Androzoo dataset) under various non-IID distributions to compare the adaptation performance of the proposed framework with other baselines FL-based malware classification model. Experimental results show that FedAdapt can maintain the highest accuracy in malware detection and classification tasks under different degrees of non-IID settings, which is superior to the state-of-the-art models, proving its best adaptive performance.

## 2. System Model and Problem Description

### 2.1. System Model

Existing deep learning-based solutions for malware classification rely on large-volume high-quality training data. However, such a training process has potential data privacy disclosure risks for Android device usersand violates existing privacy protocols [22].

To solve these privacy concerns, we introduced an FL framework and developed a privacy-protected collaborative classification model for Android malware. An FL framework allows Android clients to keep the malware dataset locally and collaboratively learn classification models, which means that any third party cannot access the user’s raw data [28]. The framework of Federated learning consists of a server and multiple clients [29]. The server in this work refers to the remote FL server, and the client is an Android client.

**Figure 1:**
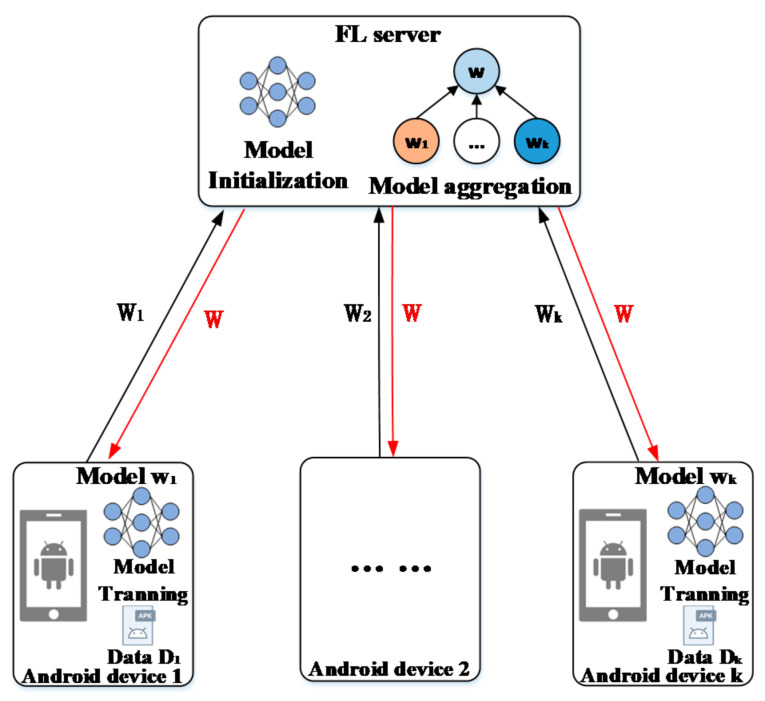
FL-based Malware classification framework.

We consider an FL-based android malware classification model that includes one FL server for parameter aggregation and update server, and m Android clients have several training samples. The FL framework uses a given training algorithm (for example, a CNN) to collaboratively train a malware classification model. The overall structure of the FL-based model is shown in Figure 1.

There are the following stages in the process of Federated learning: the initialization stage, aggregation stage and update stage, which are described as follows:

In the initialization phase, the FL Server assigns a pre-trained global model $w_{t}$. to each Android client. Then, each client trains and improves the current global model $w_{t}$ by using the local dataset $D_{k}$ of $D_{k}$ size in each iteration. 

In the aggregation phase, the FL Server collects local gradients of android node uploads. The global loss function $F\left(w\right)$. and local loss function $F_{k}\left(w\right)$. to be optimized in FL are shown in Equations (1) and (2):

(1) $$\underset{w\in R^{d}}{min}\;F(w)=\frac{n_{k}}{N}\overset{m}{\underset{k=1}{\sum }}F_{k}\left(w\right)$$

(2) $$\;F_{k}\left(w\right)=\frac{1}{n_{k}}\sum_{z_{i}\in D_{k}}^{\;}\;f\left(w;z_{i}\right)$$

where $f\left(\cdot \right)$ is the local loss function of the Android client $k,k\in \left[1,N\right]$, $z_{i}=\left(x_{i},y_{i}\right),\forall i\in \left[1,\ldots n_{k}\right]$ is sampled from the local dataset $D_{k}$ of k clients; $n_{k}$ is the number of samples of client k; N is the total number of global samples, $x_{i}\in X$ is the feature of malware, and $y_{i}\in Y$ is the category label of malware.

(3) $$w^{t+1}{}_{\;}\leftarrow w_{\;}{}^{t}+\frac{n_{k}}{N}\overset{m}{\underset{k=1}{\sum }}\nabla_{w^{t}{}_{\;}}F_{k\;}^{t}$$

In the update phase, the FL Server uses the Federated average algorithm (Fedavg) [22] to obtain a new global model $w_{t+1}$ for the next iteration, as shown in Equation (3). $\overset{m}{\underset{k=1}{\sum }}\nabla_{w^{t}}F_{k}^{t}$ denotes the model update aggregation, and $\frac{n_{k}}{N}\overset{m}{\underset{k=1}{\sum }}\nabla_{w^{t}}F_{k}^{t}$ denotes average aggregation.

This process is repeated for both the FL Server and Android clients until the global model converges. By not requiring direct access to raw training data on android nodes, this mode significantly reduces the risk of privacy disclosure.

### 2.2. Problem Description

However, the existing FL-based framework has the following problems in the task of Android malware classification:

(1) The fuzziness of Android malware makes it difficult to extract effective features.

Hackers generally use the means of avoiding detection, leading to the ambiguity of the malware features, which makes it difficult for Android clients to extract a set of effective features x for classification (on local). It will further affect the global model’s convergence efficiency and classification accuracy in federated learning.

(2) Non-IID distribution of malware on the Android clients leads to poorly training the effect of the existing FL-Base classification model:

The existing FL-Base classification model only develops a common classification model w for all clients. However, the differences in users’ (from the different organizations or communities) identities or preferences will lead to heterogeneity distribution (non-IID) between the local malware type distribution p(y), making the existing single common classification model unable to adapt to the data distribution features of each user. In other words, $E_{D_{k}}\left[F_{k}\left(w\right)\right]\neq F\left(w\right)$, the global model is difficult to converge to the optimum, and the classification accuracy is poor for local clients.

The above limitations reduce the classification effect of the FL-based android malware classification model, which prompted us to develop an adaptive multi-modal FL-based malware classification framework to achieve accurate and adaptive malware classification.

### 2.3. Abbreviations and Mathematical Symbols

The list of abbreviations and mathematical symbols used is given in Table 3.

## 3. Proposed Methods

In this section, we first introduce the composition and functions of FedHGCDroid, an adaptive multi-dimensional malware classification framework proposed by us. Then, two key modules of FedHGCDroid are introduced in detail: (1) the Multi-dimensional Android malware classification model HGCDroid and (2) the Adaptive model training mechanism, namely, FedAdapt. 

### 3.1. FedHGCdroid Framework Overview

To solve the limitations of the existing framework proposed in Section 2.2, we designed an adaptive multi-dimensional malware classification framework, involving multiple Android clients to train the malware classification model collaboratively.

#### 3.1.1. Framework of Proposed FedHGCDroid

**Figure 2:**
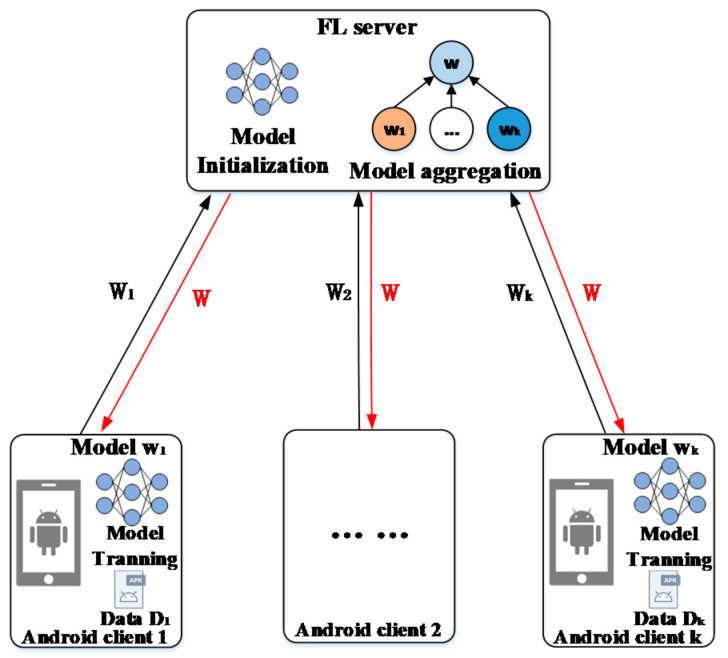
Framework of FedHGCDroid.

The system model under consideration is a federated learning framework as shown in Figure 2, which mainly comprises two types of entities, a server and k. Android clients.

(1) FL Server: An FL server with strong computing capability and rich computing resources. The FL Server contains two mechanisms: (a) initialize the global model and send the optimal global initialization model parameters to all edge clients; (b) aggregate the gradient uploaded by edge clients until the model converges.

(2) Android clients: Android clients have a few numbers of samples of various types of malware and the samples are non-IID distributed to different clients. A local classification model and adaptive Federated training mechanism are deployed on Android clients. These clients typically represent small organizations or communities, etc. 

The workflow of the framework includes the following four steps:

(1) The Android client uses the initial model paramete $\hat{w}$ of HGCDroid and the local dataset to train the adaptive classifier suitable for the local environment; 

(2) Android clients upload the update gradient ${\hat{w}}_{k}$ of initialization parameters to the FL Server; 

(3) The FL server calculates the update gradient of initialization parameters uploaded by n clients and updates the optimal global initialization parameters; 

(4) The FL Server sends new optimal global initial model parameters to each Android client. 

The above steps are repeated until the global model reaches optimal convergence. Decentralized clients can perform malware classification tasks using trained personalization models that are best suited locally.

(5) The Android clients use a trained personalization classification model to classify local malware. 

In addition, the framework of FedHGCDroid also includes two modules: (1) local Android malware classification model HGCDroid and (2) adaptive model training mechanism FedAdapt.

(1) Malware classification mechanism HGCDroid: the multi-dimensional HGCDroid model deployed in Android clients can effectively capture the behavior features of malware and classify it, which contains android malware classification knowledge from global clients.

(2) FedAdapt: The FedAdapt mechanism is deployed on the Android client and FL Server. The FedAdapt mechanism is aggregated to calculate the global initial model parameters based on the contribution degree to obtain the optimal initialization parameters. On the Android client-side, local adaptive training is carried out according to the initial model parameters shown by the cloud aggregator to obtain the most suitable local malware classification model.

#### 3.1.2. Threat Model

In the existing malware classifier training framework, it is assumed that the server is a semi-honest party who is honest in conducting all the given tasks but curious about the type and number of malicious samples (infected) owned by the clients, and the code of the app. The type and number of malicious samples (infected) owned by the client will reveal the identity and preference information of the clients 4 [15]; the source code of the app on the client-side will reveal the private information of the app (not open source) vendor.

Moreover, we assume that all clients are semi-honest, and strictly follow the designed protocols of training but may be interested in other clients’ data resources. The raw data can even be inferred from model update information.

Therefore, in this paper, we designed an FL-based malware classification framework to avoid the privacy problems existing in the traditional framework mentioned above.

#### 3.1.3. Privacy Analysis

In article [30], the author disassembled GDPR (General Data Protection Regulation) and obtained seven practical privacy compliance inspection principles: (1) Lawfulness, Fairness and Transparency; (2) Purpose of Limitation; (3) Data Minimization; (4) Accuracy; (5) Storage Limitation; (6) Integrity and Confidentiality; (7) Accountability;

We analyze the privacy compliance of the framework proposed in this paper according to these seven compliance checking principles.

(1) The principle of Lawfulness, Fairness and Transparency.

According to the principle of Lawfulness, the FL server, as the data controller, must clarify its legal basis before requiring clients to attend FL training. 

The legal basis of GDPR includes: Consent; Contract; Legal obligation; Material interest; Public task; and Legitimate interests.

The FL framework is designed in a way that does not allow FL servers to directly access raw training data to ensure privacy. The client, as a participant in the FL system, sends the results back to the FL server only when they are confirmed. In addition, FL systems only process data (local ML model parameters) for explicit purposes, which are reasonably expected by the customer and are easily regulated by specific laws. For these reasons, the laws in GDPR are suitable for implementation in this FL framework and therefore meet the principle of Lawfulness.

As for the principle of Fairness, the training method in this paper calculates the contribution of the data subject according to the data quantity and quality (accuracy) provided by the data subject and finally carries out weighted model aggregation according to the contribution. This initially ensures the fairness of their participation. As for the principle of Transparency, since the server monitors the accuracy of each client’s delivery model during each turn, it can provide the transparency of the training process appropriately. However, in order to ensure privacy, the original data set cannot be accessed except by the client itself, so a compromise is made between Transparency and privacy protection.

(2) The principle of Purpose Limitation

In this article, the FL server exposes the specific purpose of its model training to the client, who decides whether or not to participate in the training. At the same time, because the transmitted model is protected by differential privacy technology [31], the FL service provider cannot infer information from the model beyond the training task and, therefore, cannot use it for other purposes. 

Therefore, the scheme satisfies the principle of Purpose Limitation.

(3) The principle of Data Minimization

The principles of data minimization in the GDPR require the data controller (FL server) to collect and process personal data that is sufficient, limited, and only relevant to the stated purpose. In this scenario, the FL server does not need to collect and process raw training data; in contrast, the FL server only needs to collect the local ML model from the participant to aggregate the global model required by the client while being unable to infer information from the model beyond the training purpose. Therefore, the scheme satisfies the principle of Data Minimization.

(4) The principle of Accuracy

The purpose of this principle is to ensure that data controllers should keep personal data correct and not mislead any factual issues. In this article’s FL setup, the FL server updates the model only by performing weighted aggregation calculations on the models returned by the customer in terms of their contribution (accuracy). In this process, no changes are made to the customer’s model parameters, and only the model calculation results are returned. So, the scheme satisfies the principle of Accuracy.

(5) The principle of Storage Limitation

This principle states that data controllers will not hold personal data for longer if the data is no longer needed for the claimed purpose. In the FL setup of this scheme, the FL server only performs security aggregation calculations, does not store any local model parameters from participants, and the aggregated model is protected by differential privacy and anonymous processing. Therefore, the scheme satisfies the principle of Storage Limitation.

(6) The principle of Integrity and Confidentiality

In order to prevent unauthorized access, network attack, or data leakage directly from communication between the client and FL server, SSL/TLS is adopted in the data transmission process. Therefore, the scheme meets the requirements of Integrity and Confidentiality.

(7) The principle of Accountability

Because the FL server calculates and records the accuracy of the contribution of the model uploaded by the customer in each turn, the behavior of the model provider can be monitored. If the client that maliciously interferes with the training process is found, it can be traced and held accountable. Therefore, the scheme meets the requirements of Accountability. 

To sum up, the malware classifier framework proposed in this paper basically conforms to the data protection principles of GDPR.

### 3.2. Multi-Dimensional Android Malware Classification Model: HGCdroid

To solve the problem that the existing framework was not comprehensive and effective enough to mine the behaviors of Android malware, we proposed a multi-dimensional Android malware classification model HGCDroid (as a local classification model for FedHGCDroid). It combines the statistical features θ and graph features G of malware, and carries on multi-attribute coding to the API, mining the more essential behavior characteristics of malware, so as to improve the classification effect.

In this article, the classification tasks of HGCDroid are: (1) malware detection; (2) malware type classification; malware family classification malware detection.

#### 3.2.1. Sample Decompiling and Feature Preprocessing

To accurately classify malware, it is necessary to extract features that can effectively represent the behaviors of malware. The features adopted in this paper are as follows:

(1) API

API features can describe the semantic information of the app and can be used to represent the specific operations performed by malware.

(2) Permission

Permission features can describe an application’s access to sensitive resources. The hacker usually needs to apply for sensitive permissions to achieve malicious purposes.

(3) Intent filters

Intents can describe the content of communication between components. Intents can launch active components and service components. Intents can also transmit broadcast content, enabling communication between applications. Malware often uses many intents to call other applications or define Intent filters to retrieve intents broadcast by other applications that contain sensitive data.

(4) Hardware

Hardware features can describe the resources that the malware needs to access and can be used to represent the purpose of the malware.

(5) Function call diagram

The function call graph describes the application’s internal execution process and represents the application’s potential behavior. It contains a lot of semantic information and can prevent malicious applications from using obfuscation methods to a certain extent, so it has good robustness.

**Figure 3:**
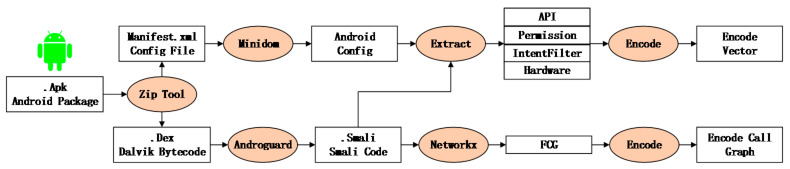
Process of sample decompiling and feature preprocessing.

The process of sample decompiling and feature preprocessing is shown in Figure 3.

(1) Use the ZIP tool to decompress the APK file to obtain the Manifest resource file, signature file, and DEX file.

(2) Use the Minidom tool to parse AndroidManifest.xml to obtain permissions, Intent filters, hardware, and other features in the configuration information.

(3) Decompile DEX file using AndroGuard tool and convert Android bytecode (class.dex) into SMALI code. The API features used in APK and the call relationships between APIs are obtained by parsing SMALI code, and NetworkX builds the function call diagram.

(4) Feature engineering screening is carried out for statistical features (API, permissions, Intent filters, hardware). The screened features are encoded through feature engineering screening according to the frequency of occurrence. The encoded statistical feature vector is used as the input of the CNN module to mine the implicit relations in statistical features.

(5) The function call graph (including node vector and adjacency matrix) was constructed as the input of the GNN module. Through the call relationship between function nodes, the hidden behavior in malware can be mined.

#### 3.2.2. Multi-Attribute Coding Method for APIs

The API nodes in the function call graph have many available attributes, but the existing research only uses semantic attributes, resulting in the limitation of the information that can be used in the subsequent GNN model. In order to enrich the information contained in the function call graph, semantic attribute, functional attribute, permission attribute, and frequency attribute of the API node are used to encode. Meanwhile, we use the Word2Vec [32] method to encode semantic attributes, functional attributes, and permission attributes, respectively, in order to reduce the computation amount. Compared with the one-hot encoding in the existing scheme, it can:

(1) Reduce the dimension of coding vector G, and capture the functional similarity between different versions of APIs;

(2) Enrich the malicious behavior information of function call graph;

(3) Improve the anti-time attenuation ability of the classifier;

(4) Improve the robustness of extracting features against malware confusion to a certain extent. 

The proposed multi-attribute coding method for API consists of the following four parts:

(1) Semantic attribute refers to the meaning represented by the API node itself in the context of the application program. The application program is regarded as a document in natural language processing. Different orders of API occurrence may indicate different intentions and behaviors. Semantic encoding mainly uses the textual semantic information of APIS for embedded encoding. The APIs that appear in the context at the same time have similar or similar coding vectors. 

(2) Function attribute refers to the function cluster to which the API belongs. The package and class to which the API belongs are used to represent the function to which the API belongs. According to the characteristics of the Android API framework, APIs belong to specific packages and classes, and all APIs in the same package and class have similar functions. According to these characteristics, this paper encodes the API using the names of the packages and classes it belongs. 

(3) Permission attribute refers to the permission to apply for using the API. According to the Android permission management mechanism, access to some APIs requires specific permission, and permission will also restrict a group of specific sensitive operations. In this paper, the permission belonging to API is regarded as the permission attribute of API, and the permission code is obtained by embedding the permission with the text semantic information of the permission. 

(4) Frequency attribute represents the frequency of the API node appearing in the current application and is the coding content related to the current application.

We define $st\left(v\right)$, $ft\left(v\right)$, $pm\left(v\right)$, $tm\left(v\right)$ to represent the encoding methods of the semantic attribute, functional attribute, permission attribute, and frequency attribute, respectively, and finally obtain the attribute code $\varphi \left(v\right)$ of API node, as shown in Formula (4).

(4) $$\varphi \left(v\right)=\left[st\left(v\right),ft\left(v\right),pm\left(v\right),tm\left(v\right)\right],v\in V$$

#### 3.2.3. Network Architecture of HGCDroid

**Figure 4:**
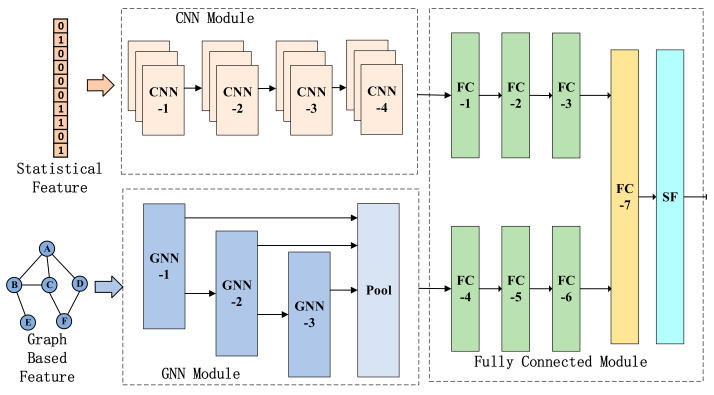
Multi-dimensional malware classification model HGCdroid.

The network architecture of HGCDroid includes an input layer, GNN model based on graph neural network, CNN model based on convolutional neural network and fully connected module, and output layer, as shown in Figure 4. The input of HGCDroid is mainly the coding vector θ of statistical features and the coding function call graph G. Function call graph G is a description of the internal execution process of the application and represents the potential behavior of the application. It contains a lot of semantic information and can prevent malware from using obfuscation methods to a certain extent, so it has good robustness. The coding vector θ of statistical features is used as the supplement of graphical features G, so as to capture malicious behavior features more comprehensively and improve the accuracy of classification.

The CNN module is used for processing statistical features of Android malware, mining hidden relations in statistical features through convolution operation, and embedding statistical features into low-dimensional vectors for output. The GNN module is used to process the function call graph features, obtain the dependency between function nodes through graph convolution operation, learn the structural features of the function call graph, and finally generate the embedded representation of the function call graph. The fully connected module is used for receiving the embedding vector output by the CNN module and the GNN module, mining the combinatorial relations in embedded features through the fully connected layer and establishing the mapping between embedded features and output to complete the final classification. The following contents, respectively, introduce the input features and the composition of the CNN module, GNN module, and full connection module. 

(1) CNN module

The input of the CNN module is statistical feature θ, which is composed of the permission feature, API feature, Intent filter feature and hardware feature in the order of semantic relationship. Meanwhile, each feature is encoded according to the frequency of its occurrence. 

CNN module mainly includes a convolution layer, normalization layer, RELU layer, pooling layer, etc., and extracts specific patterns [33] and hidden information in statistical feature through convolution units composed of different neural network layers, and finally output the expanded one-dimensional vector.

In this paper, the one-dimensional convolution operation is used as the convolution layer, and Formula (5) gives the specific calculation method. The input statistical features of the module are encoded according to the specific order of statistical feature vectors to obtain the one-dimensional feature vector HGCdroid. Since different features are segmented encoded according to the character order, there are similarities between adjacent features, so the convolution operation is used to obtain the combined relations between different features. The speed of convolution operation can be greatly improved by using the features of weight sharing and local perception.

(5) $$y_{j}=f(b_{j}+\underset{i}{\sum }k_{ij}*x_{i})$$

RELU layer can make the output of some neurons turn to zero, make the network become more sparse, reduce the interdependence between parameters of the same layer, and effectively alleviate the over-fitting problem in deep learning. Table 4 shows the network structure and parameters of the CNN module.

(2) GNN module

The GNN model input coding module function call graph $G=\left(V,E\right)$, V is the node-set of the function call graph, E is the edge set of the function call graph, it contains a convolution layer, RELU layer, the global pool layer, etc., to form the figure convolution unit, a function call graph and embedded vector mapping relationship, mining function calls the structure of the information hidden in this picture. Finally, the one-dimensional vector is the output. The GNN module firstly carries out information dissemination, sampling function nodes around, $N\left(s\right)$ means computing all neighbor nodes, including the current node, multiplying feature vectors of neighbor nodes by weights, and sending them to adjacent function nodes, as shown in Equation (6). Then, information aggregation is carried out to fuse the information of the current node with the information of the surrounding nodes, and the vector representation of the current node is obtained through the transformation of the nonlinear activation function, as shown in Equation (7).

(6) $$N\left(s\right)=\{u|\left(u,v\right)\in E\}$$

(7) $$h_{v}^{k}=\sigma \left(W_{1}^{k}\cdot h_{v}^{k}+\underset{u\in N_{L}\left(s\right)}{\sum }h_{u}^{k}\cdot W_{2}^{k}\right)$$

The GNN module adopts the same activation function as the CNN module, the RELU function, which is used to introduce nonlinear changes and certain sparsity to enhance the expression ability of the model. The Readout layer splices the embedding vector Readout by two global pooling functions into the final graph embedding vector. Table 5 shows the network structure and parameters of the GNN module.

(3) Fully connected module

The fully connected module processes the feature vectors output by the CNN module and GNN module and captures the combined relations between embedded features to achieve the final classification goal. The output of a fully connected neural network is adjusted according to different tasks. For a malicious software detection task, its output is a 2-dimensional vector corresponding to the probability of malicious software and benign software, respectively. For the classification task of malware types, the output is an 8-dimensional vector, corresponding to the probability of 8 types of malware and benign software, respectively. For the malware family classification task, the output is a 10-dimensional vector, corresponding to the probability of 10 different malware families. Fully connected modules include Linear, LayerNorm, LeakyRelu, and Softmax layers.

The LayerNorm layer is used to normalize the features within a single sample, which can eliminate the distribution bias of input features and retain the distribution characteristics of input features. The LeakyRelu layer is used in this paper to establish a non-linear mapping, and Formula (8) gives the calculation method.

(8) $$\mathrm{LeakyRelu}\left(x\right)=\left\{\begin{array}{cc} x & x>0\\ \alpha \left(e^{x}-1\right) & x<0 \end{array}\right.$$

In this paper, the output of the CNN module and GNN module are, respectively, processed by a three-layer fully connected unit, and the output vector of the model is obtained by the Softmax function. Each value of the output vector represents the probability corresponding to each category. 

In this paper, the cross-entropy function is mainly used to calculate the loss between the output vector and the label, as shown in Formula (9). Where $y_{i}$ represents the label of category i, $p_{i}$ represents the prediction probability of category i output by the model, K represents the number of categories, and $J_{HGCDroid}$ represents the loss function of the model.

(9) $$J_{HGCDroid}=-\overset{K}{\underset{i=1}{\sum }}y_{i}\log\left(p_{i}\right)$$

Finally, the linear layers of the two modules are added together, and the final output is calculated by the Softmax function. Each value of the output vector represents the probability of the corresponding category. Table 6 shows the network structure and parameters of the fully connected module, and the output size is determined by different classification targets.

### 3.3. Adaptive Training Mechanism of Classification Model Based on Contribution Degree: FedAdapt

To solve the problem of poor adaptability of the existing FL-based framework to non-IID distribution, this paper innovatively improves the existing Fedavg algorithm by introducing meta-learning and attention mechanisms and designs an adaptive model training mechanism based on contribution degree, namely, FedAdapt. 

As described in the problem description section in Section 2.2, existing popular FL schemes have poorer classification accuracy in higher degree non-IID scenarios. Because in a higher degree of non-IID scenario, local model parameters of different clients will deviate more seriously, resulting in a decline in the aggregation effect of global model parameters and a sharp decline in the final classification accuracy.

Therefore, in this paper, we define the adaptability of the FL framework as its ability to maintain classification accuracy in different degrees of non-IID scenarios.

The improvements of FedAdapt include two aspects: (1) in the training process of the local model, we introduce a first-order learning method (reptile [34]), which is used to extract more generalized general features between different clients (different distributions) to reduce the overfitting of the local distribution; (2) in the process of aggregation of model parameters, we introduce an attention-based mechanism to calculate the contribution of different local models to the global model (based on classification accuracy) and improve the aggregation effect of local models with different qualities in non-IID scenarios.

#### 3.3.1. Meta-Learning-Based Local Model Training Method

The meta-learning method can learn multiple internal representations of different tasks and has the advantage of strong generalization ability for new tasks. Therefore, we consider the meta-learning method to improve the existing Federated learning model training method and improve the generalization of shared model parameters, so as to solve the problem of adaptability of the existing framework to the non-IID distribution of malware on clients.

Inspired by the meta-learning algorithm, we introduced the initial model parameters calculation method in the meta-learning algorithm into the existing FL framework for improvement, and the android malware dataset on the client is regarded as multiple different training tasks. This model training method aims to train initial model parameters with strong generalization capability for all clients, which can be a slight adjustment by malware data of clients to train an adaptive classification model adapted to the distribution of malware on clients.

It should be emphasized that the generalization ability of initial model parameters has been proved in the article [34]. It is pointed out that the initialization parameters, which are found in the meta-learning approach, are close to all of the optimal solution manifolds of the training tasks, as shown in Equation (10).

(10) $$\underset{\hat{w}}{min}D_{Euclidean}\left(\hat{w}\;,{\tilde{w}}_{k}^{*}{}_{\;}\right)$$

where $\hat{w}$ is the initial model parameters, and ${\tilde{w}}_{k}^{*}$ denotes the set of optimal parameters for task k. The goal is finding $\hat{w}$ such that the Euclidean distance $D_{Euclidean}\left(\hat{w}\;,{\tilde{w}}_{k}^{*}{}_{\;}\right)$ is small for all clients (tasks).

**Figure 5:**
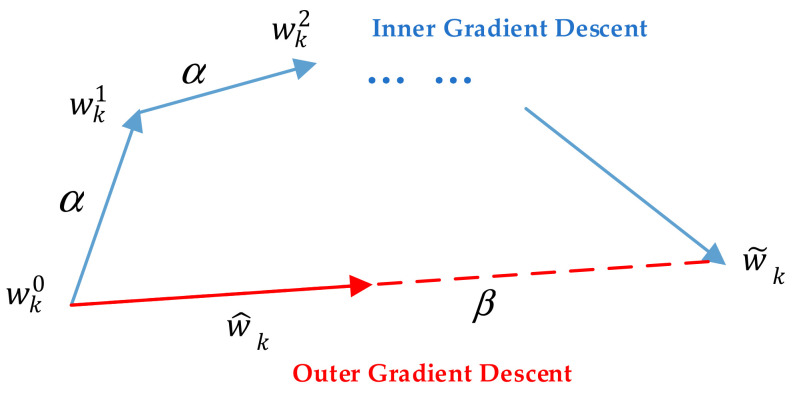
Schematic diagram of model training method based on meta-learning.

The initial model parameters $\hat{w}$ training method is divided into inner and outer gradient descent. First, inner gradient descent is performed at an inner learning rate α using the malware dataset on the client. Then, the outer gradient descent is carried out using the model weight variation value after training in the local malware dataset using the outer learning rate β, as shown in Figure 5.

Equations (11)–(13) give the calculation methods of inner and outer gradient descent, respectively. Where $w_{k}^{t}{}^{\;}$ is the weight of the model after the t rounds of the inner gradient descent on client k, and $\nabla F\left(w_{k}^{t}\right)$ is the derivative of the loss function of the model to the weight of the model. The model weight $w_{k}^{t+1}$ of the next round of inner gradient descent is obtained by iterative Formula (12). ${\tilde{w}}_{k}$ is the result of local multi-round inner gradient descent, and $\hat{w}$ is obtained by the global training of the first round. Outer gradient descent is the final result of local model training ${\hat{w_{\;}}}_{k}$ based on model initialization parameters.

(11) $$w_{k}^{0}={\hat{w}}_{\;}{}^{\;}$$

(12) $$w_{k}^{t+1}\leftarrow w_{k}^{t}-\alpha \nabla_{w}F\left(w_{k}^{t}\right)$$

(13) $${\hat{w_{\;}}}_{k}{}_{\;}\leftarrow w_{k}^{0}+\beta \left({\tilde{w}}_{k}-w_{k}^{0}\right)$$

Then, global average aggregation is carried out to update the initial model parameters ${\hat{w}}_{\;}$, as shown in Equation (14).

(14) $${\hat{w}}_{\;}{}^{\;}=\frac{n_{k}}{N}\overset{m}{\underset{k=1}{\sum }}{\hat{w_{\;}}}_{k}{}^{\;}$$

After several rounds of global training, the optimal shared initial model parameters are obtained. Each user can obtain the optimal local model ${\tilde{w}}_{k}^{*}$ by gradient descent according to Equation (3) based on the initial model parameters $\hat{w}$.

#### 3.3.2. Contribution Degree-Based Model Aggregation Method

In the FL framework, the dataset’s quality varies from client to client, and the hacker may even put false data. Therefore, the dataset on the client has different effects on the accuracy of the global model. However, the existing framework simply adopts the quantitative ratio as the weighted basis for aggregation, which makes it difficult to effectively utilize the information of each client, thus resulting in a poor global model effect. Therefore, the gradient contribution of each client needs to be quantified further to improve the effectiveness of the aggregation of the model.

From the perspective of model aggregation and inspired by the attention mechanism, this paper proposes a method to measure the contribution degree of the client, judging the merits and demerits of the local model provided by the client according to the situation of each aggregation. First, the accuracy change value Δacc of the model is defined to represent the model improvement in this update. The projection $\rho_{k}^{t}$ of client model update vector on the aggregated model update direction is regarded as the contribution of client k to model update in round t. 

If the accuracy is improved and the update direction of client k is consistent with that of the server, it indicates that the model provided by the current client has a high contribution, which improves the model accuracy, the contribution degree of this round is positive. Suppose the accuracy rate decreases and the update method of client k. is consistent with the update direction of the server; in that case, it means that the model provided by the current client leads to a decrease in the accuracy rate of the model, and its contribution of this round is negative. Calculation method of $\rho_{k}^{t}$, as shown in Equation (15).

(15) $$\rho_{k}^{t}=\Delta acc^{t}\frac{\left(w_{k}^{t}-w^{t-1}\right)\cdot \left(w^{t}-w^{t-1}\right)}{\left|w^{t}-w^{t-1}\right|}$$

According to the total number of clients’ contributions to each round of training, ${\tilde{\rho }}_{k}^{t}$ of the current client’s historical contribution is estimated, which objectively describes the data quality of the client and the total influence of the update effect of the client model on the whole model. Calculation method of ${\tilde{\rho }}_{k}^{t}$, as shown in Equation (16).

(16) $${\tilde{\rho }}_{k}^{t}=\overset{t}{\underset{i=1}{\sum }}\rho_{k}^{i}$$

Finally, with the process of model training, the weight of each client model aggregation is automatically adjusted according to the historical contribution of the client, and the local model aggregation is adaptive. Equation (17) gives the calculation method of model weight. Firstly, the sigmod formula is used to compress the historical contribution degree, and the value of the historical contribution degree is converted to between 0 and 1. Then, the weight of the current client model update $\eta_{k}^{t}$ is calculated, which calculates the weight of all models in this update round based on their historical contribution.

(17) $$\eta_{k}^{t}=\frac{sigmod\left({\tilde{\rho }}_{k}^{t}\right)}{\sum_{k=1}^{N\;}sigmod\left({\tilde{\rho }}_{k}^{t}\right)}$$

It automatically adjusts the weight of each client during model aggregation based on the client contribution. The ability to take full advantage of clients with lots of high-quality data enables clients that provide high-quality data training for local models to have greater model aggregation weights. In addition, malicious clients will be excluded to a certain extent. For example, a client that provides fake data will obtain a smaller model update weight and have a smaller impact on the global model due to a smaller contribution from the calculation. In essence, it is a process of feedback adjustment. Each iteration will adjust the process of the next round of aggregation according to the test accuracy Δacc and model updating weight feedback from the client. At the same time, this mechanism does not bias the model to a particular client’s data distribution, because Δacc ensures a higher contribution to the local model with good results on all test datasets.

When aggregating on the FL Server side, consider the cumulative contribution of the client. The aggregation of weights is realized by taking the cumulative contribution degree as one of the indicators of aggregation. Finally, the contribution-based model updating algorithm is shown in Equation (18):

(18) $${\hat{w}}_{\;}=\eta_{k}^{t}\overset{m}{\underset{k=1}{\sum }}{\hat{w}}_{k}$$

#### 3.3.3. The Training Process of FedAdapt

**Figure 6:**
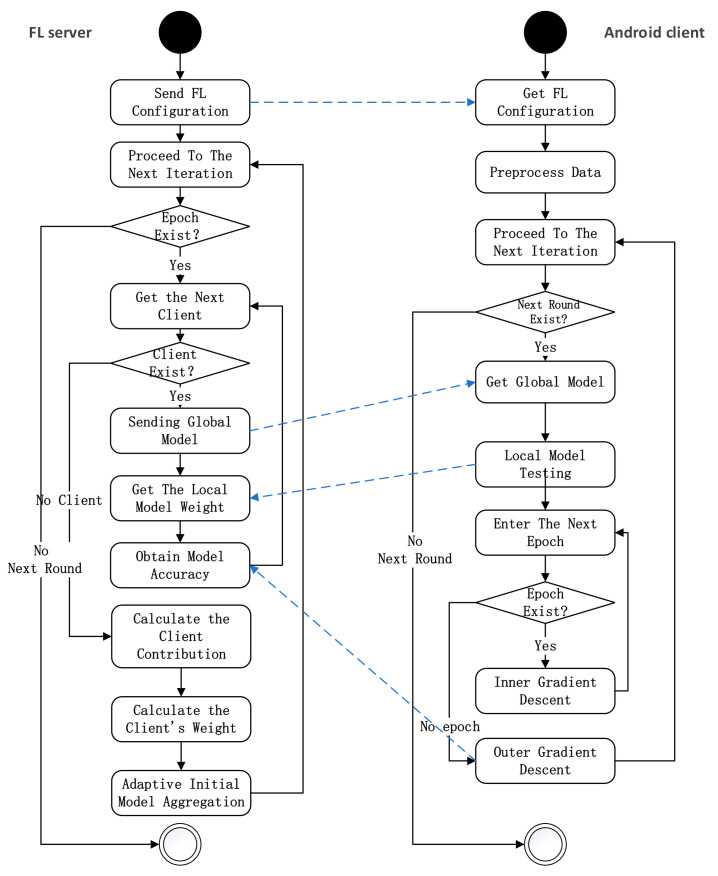
Process of adaptive classification model based on contribution degree.

The adaptive model training mechanism based on contribution is shown in Figure 6. The specific process is as follows:

(1) Global initialization: first, initiate the request of Federated learning on the server, and establish a communication link between the server and the client. The client downloads feature extraction tools and configuration parameters from the server, preprocesses data, divides the dataset into training sets and test sets, and extracts the features of malware. The server and client complete global initialization.

(2) Model download: the server sends model parameters to all clients. The client loads the global model.

(3) Model training: the client regards local model training as an independent task in meta-learning. According to the inner learning rate α, the inner gradient descent is applied to the training set to obtain the model weight ${\tilde{w}}_{k}$. The outer layer learning rate β is used for outer gradient descent and learning is performed on the initial model parameters $\hat{w}$. The locally updated weight of the ${\hat{w}}_{k}$ can be obtained.

(5) Model aggregation: the server calculates the weight of each client according to the change of model accuracy and the client-server according to the historical contribution of the client, and aggregates the client model to obtain a new global model.

(6) Model test: the server sends the new global model to each client. The client tests the training dataset to obtain the accuracy of the global model, which is used to measure the local performance of the global model. According to the change value of model accuracy and weight of local model update, the server calculates the contribution of all clients in this round and adds it to the historical contribution.

(7) Repeat the training process of Steps 2–6 until the model converges, as shown in Algorithm 1. Training mechanism of adaptive model based on contribution degree. In the actual training process, model testing can be combined with model training and the model aggregation process without increasing the number of communication rounds.
**Algorithm 1: Training mechanism of adaptive classification model based on contribution degree.****Input**  
Number of iterations T, number of clients N, number of iterations of client dataset E, batch of client data B, weight of client k in round t is $w_{k}^{t}$, client learning rate α, **Output**  
Vector representation of a node $z_{v},\forall v\in V$
1:function ServerAggregate:
2:  for t=0,1,…,T do3:    Send global model $w^{t}$ to each client4:    for k=0,1,…,N do5:       
$w_{k}^{t+1}\leftarrow ClientUpdate\left(k,w^{t}\right)$
6:       
$acc_{k}^{t+1}\leftarrow ClientTest\left(k,w^{t}\right)$
7:    end for8:    
$\Delta acc^{t+1}\leftarrow \frac{1}{N}\sum_{k=1}^{N}acc_{k}^{t+1}-acc^{t}\;$
9:    $\rho_{k}^{t+1}=\Delta acc^{t+1}\frac{\left(w_{k}^{t}-w^{t-1}\right)\cdot \left(w^{t}-w^{t-1}\right)}{\left|w^{t}-w^{t-1}\right|}$10:    
$\eta_{k}^{t+1}=\frac{sigmod\left({\tilde{\rho }}_{k}^{t}\right)}{\sum_{k=1}^{N\;}sigmod\left({\tilde{\rho }}_{k}^{t}\right)}$
11:    
$w^{t+1}\leftarrow \overset{N}{\underset{k=1}{\sum }}\eta_{k}^{t+1}w_{k}^{t+1}$
12:  end for13:end function14:function $ClientUpdate\left(k,w^{t}\right)$:15:  Initialize local model weights $w_{k}^{t}\leftarrow w^{t}$
16:  for e=1,2,…,E do17:    for b=1,2,…,B do18:       
$\;w_{k}^{t}\leftarrow w_{k}^{t}-\alpha \nabla_{w}F\left(w_{k}^{t}\right)$
19:    end for20:    ${\tilde{w}}_{k}\leftarrow w^{t}+\beta \left({\tilde{w}}_{k}-w^{t}\right)$
21:  end for22:  return ${\hat{w}}_{k}$
23:end function

## 4. Results

In this section, Section 4.1 introduces the experimental data set, experimental environment, indicators, and simulation scenarios (partition method) for the non-IID distribution of the malware on the clients.

Then, the proposed scheme is compared and verified in Section 4.2, which includes the following parts:

(1) Compare and analyze the performance of the proposed client local classification model HGCDroid and the existing schemes in malware detection and classification tasks. Classification performance indicators include accuracy, precision, recall, and F1-score.

(2) The performance of FedHGCDroid was compared and analyzed with the existing FL-Base scheme in the task of malware detection and classification. The performance comparison includes two aspects: classification performance and adaptive performance. The classification performance index includes accuracy, which is compared in the IID scenarios (the degree of non-IID is close to 0); Adaptive performance is measured by the ability to maintain classification accuracy under different non-IID scenarios.

### 4.1. Simulation Setup

#### 4.1.1. Dataset

To verify the validity of the model and algorithm, we build the dataset from the open-source project of the AndroZoo [35], about 70,000 applications for malware detection and classification tasks.

In order to continue to study the types and families of malware and provide a more detailed description of malware, we used the VirusTotal [36] aggregation engine test platform to generate a simple report of malware, and based on the research results of Euphony [37], add tags for consistent high confidence malware type categories and family categories. The following Table 7 describes the malware dataset.

In the literature [38], the authors point out that spatial bias will be caused by distributions of training and testing data that are not representative of a real-world deployment, and the rate of malware in Android in the real environment is roughly 1/10.

Therefore, in order to eliminate the space bias, we set the malware-to-goodware ratio in both training and testing of each task to be 10:1. We set the ratio of training-to-testing to be 8:2. 

At the same time, in order to eliminate the time bias, we split the training set and data set by time. According to the method of reducing time bias mentioned in the literature [39], a small portion (2%) of representative (over different periods of time) Android malware has been selected for training, respectively, in the client samples using active learning.

**Figure 7:**
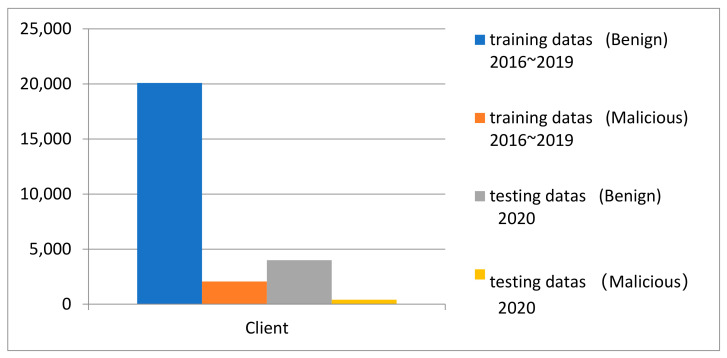
Partition result of training set and test set for centralized training.

The partition result of the training set and testing set for centralized training is shown in Figure 7. 

**Figure 8:**
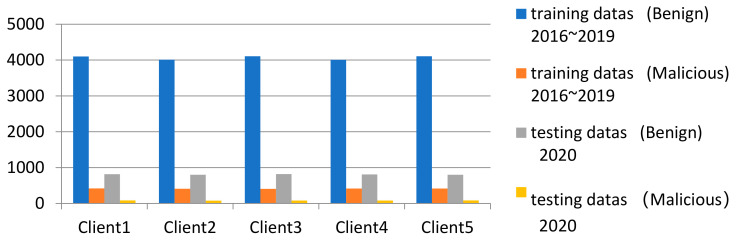
Partition result of training set and test set for FL training.

In the federal training task, the training and test sets separated by time are further divided into each client, and each client uses its own training set and test set, respectively, for task testing. The partition result of the training set and test set for FL training is shown in Figure 8.

#### 4.1.2. Experimental Environment

The software and hardware environment of experiments are shown in Table 8.

#### 4.1.3. Performance Metrics

To compare the effectiveness of the deployed models, we rely on standard machine learning performance metrics such as accuracy, precision, recall, and F1-score, as shown in Table 9. 

Malware detection is a binary classification task, and we define the malicious sample as positive and the benign sample as negative. For multi-classification evaluation indicators, we define each category is regarded as a positive sample and the other categories as a negative sample. Then, the average value of evaluation indicators of all categories is taken as the final evaluation indicator. 

#### 4.1.4. Non-IID Data Partitioning and Non-IID Degree Measurement Methods

In order to verify the adaptive performance of the proposed FedHGCDroid in non-IID scenarios, we designed a variety of non-IID malware data partitioning and measurement methods and verify their validity.

- Setting method of malware’s distribution on the client;

In order to verify the adaptive performance of the FL-based malware detection method under the non-IID situation, the centralized dataset is divided into several non-IID data subsets. We have implemented a variety of different data partitioning methods, and different data partitioning results represent different data distribution types, which will have different influences on the FL algorithm.

In this paper, the most common label distribution skew is tested. According to different data partitioning methods $P_{m}^{\gamma }$, as shown in Equation, the dataset D is divided into a set of data subset D, which follows different data distribution D. Where m is the name of the data partitioning method, γ is the scale parameter of the data partitioning method, and n is the number of clients, that is, the number of data subsets.

(19) $$P_{m}^{\;\gamma }:D\to \left\{D_{1},D_{2},\ldots ,D_{n}\right\}$$

We have implemented a variety of data partition methods with different distributions, including independent identically distributed(IID) data partition $P_{iid}$, $P_{num}^{\gamma }$ based on quantity, $P_{dir}^{\gamma }$ based on Dirichlet distribution, $P_{exp}^{\gamma }$ based on exponential distribution. Dataset D can be divided into different data subsets $\left\{D_{1},D_{2},\ldots D_{n}\right\}$.

**Figure 9:**
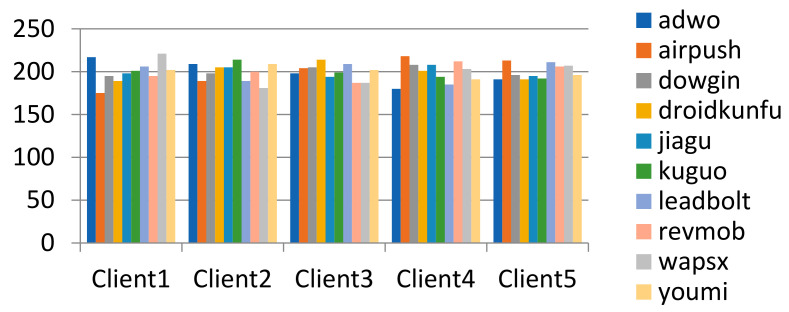
Data division result by method based on independent and identically distributed data partitioning $P_{iid}$.

(1) Independent and identically distributed data partitioning method $P_{iid}$:

The original data samples were randomly shuffled, and the data set was randomly divided according to the number of data subsets. The method can generate independent and identically distributed data subsets, and the number of samples of each label in each data subset is similar. The data partitioning results of the malware family classification task are shown in the figure. (It should be emphasized that the data set on each client will be further divided into the training set and test set according to the time-split method described above). The data partition result on the clients as shown in Figure 9.

**Figure 10:**
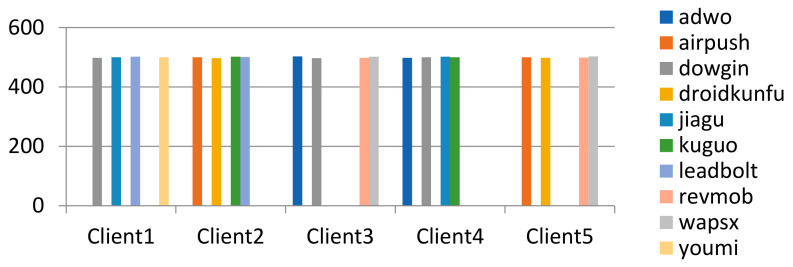
Data division result by method based on the number of labels $P_{num}^{\gamma }$ (γ = 4).

**Figure 11:**
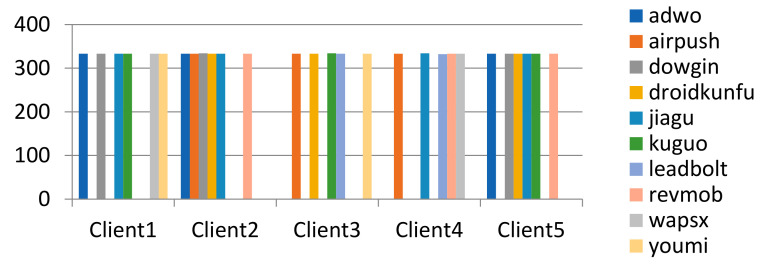
Data division result by method based on the number of labels $P_{num}^{\gamma }\;$ (γ = 6).

(2) Data division method based on the number of labels $P_{num}^{\gamma }$: 

Each data subset firstly selects a fixed number of labels, and then randomly selects samples under its labels. The method can make different subsets of data have different samples of labels, where γ represents the number of labels in a single data subset. Taking γ value = 4 and 6, the data partition result on the clients is shown in Figure 10 and Figure 11.

**Figure 12:**
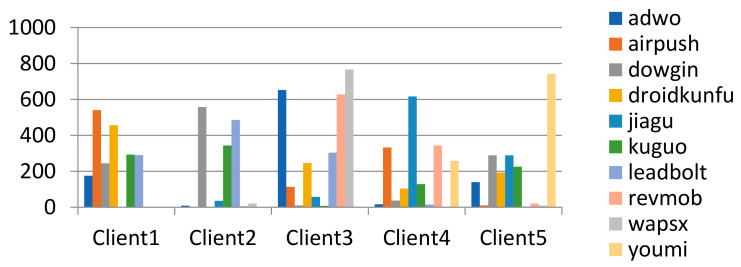
Data division result by method based on exponential distribution $P_{exp}^{\gamma }$ (γ = 0.4).

(3) Data partitioning method based on Dirichlet distribution $P_{dir}^{\gamma }$: 

Dirichlet distribution represents the distribution of distribution and can adjust parameters to obtain data subsets of different distributions. The method can produce subsets of data that follow the Dirichlet distribution, where γ represents the parameters of the Dirichlet distribution. If γ is set to a smaller value, then the partition is more unbalanced. Take γ value = 0.4, the data partition result on the clients as shown in Figure 12.

**Figure 13:**
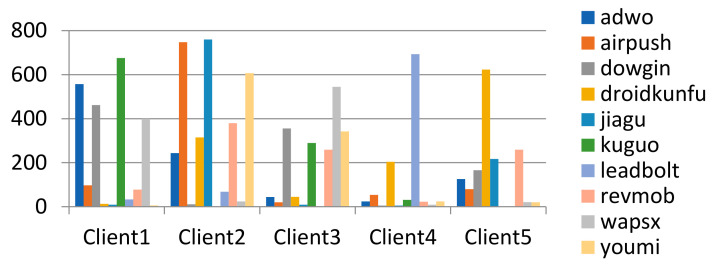
Data division result by method based on exponential distribution $P_{exp}^{\gamma }$ (γ = 10).

(4) Data division method based on exponential distribution $P_{exp}^{\gamma }$: In order to increase the distribution difference between data subsets, the samples of each label are divided according to random values generated based on exponential distribution. This method can obtain a subset of data based on exponential distribution, where *γ* represents the scale of exponential distribution change. If γ is set to a smaller value, then the partition is less unbalanced. Take γ value = 10, the data partition result on the clients as shown in Figure 13.

- The method of quantifying the degree of non-IID in the distribution of malware on clients;

We propose a method for quantifying the degree of non-IID in different data partitioning methods, as shown in Equation

(20) $$d\left(D_{1},D_{2}\right)=\frac{1}{2}\overset{N}{\underset{i=0}{\sum }}\left|P\left(y_{i}|D_{1}\right)-P\left(y_{i}|D_{2}\right)\right|$$

where N represents the number of labels and $P\left(y_{i}|D_{1}\right)$ said in a data subset $D_{1}$ label $y_{i}$ in probability. 

For a subset of data $\left\{D_{1},D_{2},\ldots D_{n}\right\}$, the distance of distribution between any two data subsets represents the degree of non-IID, as calculated in Equation.

(21) $$\tau (\left\{D_{1},D_{2},\ldots D_{n}\right\})=\frac{2}{N(N-1)}\overset{i<N,j<N}{\underset{i<j}{\sum }}d\left(D_{1},D_{2}\right)$$

It is a symmetrical distribution difference calculation method that does not cause too much oscillation due to the occurrence of zero value.

- Client’s malware distribution setting and effect verification of distribution difference quantification method

To verify the validity of the method for the data partitioning and the method for quantifying the degree of non-IID in the distribution of malware on clients, we carried out experiments on the dataset of malware family classification. The data were divided into ten subsets by different partitioning methods, and non-IID’s degrees of them were calculated.

**Figure 14:**
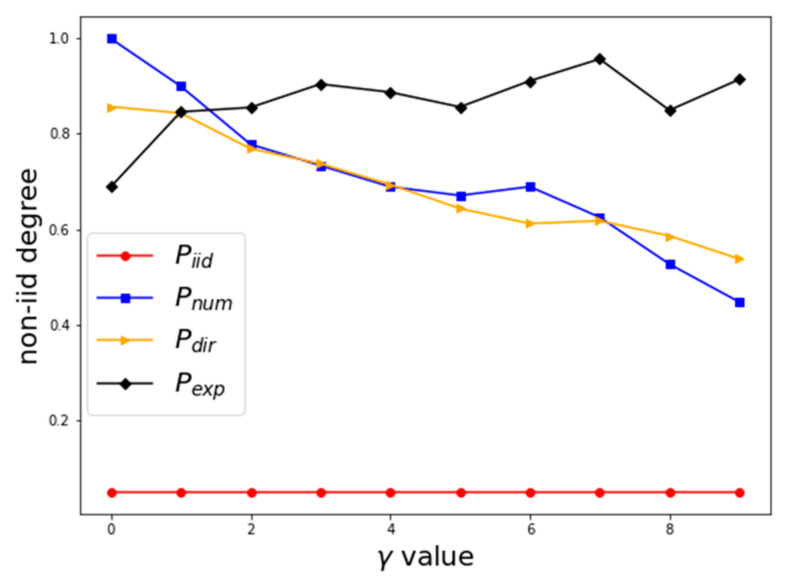
Client’s malware distribution setting and effect verification of distribution difference quantification method.

The experimental results are shown in the client malware distribution setting and effect verification of distribution difference quantification method. The abscissa represents the parameter controlling the distribution difference, and the ordinate represents the calculated value of the label distribution difference. With the value of γ increasing, the non-IID’s degree of $P_{dir}$ and $P_{num}$ are decreasing gradually, and the non-IID’s degree of $P_{exp}$ is increasing, as shown in Figure 14.

Experimental verification shows that different data partitioning methods can generate multiple types of non-IID data and simulate different scenarios of non-IID distribution of malware data. The method of quantifying the degree of non-IID can clearly describe the degree of distribution difference of data partitioning methods and provide an intuitive quantitative description.

### 4.2. Comparison and Analysis of the Results of Experimental Tasks

#### 4.2.1. Test Classification Performance of HGCDroid and Other Malware Classification Model

To verify the effectiveness of the HGCDroid (local model of FedDroid) model in this paper, extensive comparative experiments were conducted, including two experimental tasks: malware detection and malware type classification (Train in a centralized method).

We compare the effectiveness of our approach with four state-of-the-art works, including SVM (Arp et al. 2014) [12], DNN (Jiang et al. 2018) [18], CNN (Taheri et al. 2021) [23], GNN (Pektas et al. 2020) [19] with an identical simulation configuration. 

SVM (Arp et al. 2014) [12] and CNN (Taheri et al. 2021) [23] applied the statistical-based features, and DNN (Jiang et al. 2018) [18] and GNN (Pektas et al. 2020) [19] applied the Graphical-based features. The experimental results are shown in Table 10.

As can be seen from the results given in Table 5, in the classification task of malware type, the proposed classifier HGCDroid has a great advantage in Accuracy, with an increase of about 3%. This is because the API multi-attribute coding method and multidimensional model structure (CNN and GNN) in HGCDroid can mine the behavior characteristics of malware more effectively, which makes a more accurate distinction between different types of behavior. Meanwhile, Precision, Recall and F1-score all reached the highest.

In the detection task of malware, the proposed classifier HGCDroid achieves the highest Accuracy, Recall and F1-score, while its precision is slightly lower than DNN (Jiang et al. 2018) [18] (DNN is used to mine the graph features). It shows that the multi-dimensional mining of malicious behavior has no obvious advantage in the binary classification task. However, in multi-type classification, multi-dimensional mining of malicious behavior has greater advantages.

#### 4.2.2. Test the Performance of the FedHGCDroid and Other FL-Based Malware Classification Schemes under Different non-IID Data Settings

In this section, we compare the performance of our proposed FedHGCDroid scheme with some state-of-the-art studies on FL-based malware classification schemes including Fed-IIoT (Taheri et al. 2021) [23], RAPID (Shukla et al. 2021) [22], LiM (Galvez et al. 2021) [21] with an identical simulation configuration. We fully reproduce these malware classification schemes in our work and compare the performance (classification performance and adaptive performance) with the proposed FedHGCDroid. For a more comprehensive comparison of the proposed schemes, we also combine the existing advanced FL algorithm (such as Fedavg, Fedper [40], Fedamp [41]) with the proposed HGCDroid model as a comparative experiment.

To compare the performance of different algorithms, this paper carries out experiments on the three tasks of malware detection, type classification, and family classification, respectively. In order to compare the performance of algorithms under different data distribution scenarios, this paper selects five types of data distribution settings for comparative experiments to verify the performance.

All algorithms take the same federated learning settings. A total of five clients were set, with iterations of 500, a client epoch of 5, a batch size of 128, a learning rate of 0.01, an optimizer of SGD and a learning rate of 0.01.

The experimental results are shown in Table 11, Table 12 and Table 13, with each column representing different data partitioning methods and each row representing different FL-based malware classification schemes. Fed-IIoT [23], RAPID [22], LiM [21] and [Fedavg + HGCdroid] are four kinds of classification schemes based on global model training methods. After training, a globally unique model is generated and sent to different clients for testing. 

[Fedamp + HGCdroid], [Fedper + HGCdroid] and our proposed FedHGCdroid are three personalized federated learning and training methods. After accepting the global model, their local models adjust appropriately according to the distribution of local data. The results of the experiment are shown and analyzed below:

- Comparison of classification performance between FedHGCDroid and other state-of-the-art studies on FL-based malware classification schemes

In order to compare the classification performance of respective FL-based malware classification schemes, we compared the classification effect under the condition of IID. It should be pointed out that in the IID case (ideal case), the degree of non-IID is close to zero and the distribution of clients is almost the same, not affected by degradation after model aggregation, which can fairly compare the classification performance of different schemes. 

As shown in Table 11, Table 12 and Table 13 (“IID” in the first column), FedHGCDroid achieves the highest classification accuracy in malware detection and malware family classification; however, in the task of malware classification, the classification accuracy is slightly lower than [Fedavg + HGCdroid].

This illustrates two conclusions: under ideal conditions (IID), FedAdapt, the adaptive mechanism in FedHGCDroid, is not significantly different from Fedavg, the existing FL aggregation algorithm (because they both use the same HGCdroid as the local classifier).

At the same time, compared with the existing FL-based malware classification model, our classification performance is the highest in all three malware tasks. This is because we adopt the multi-dimensional malware classification model HGCDroid as the local classification model, and their schemes only use relatively simple statistical features, which cannot effectively mine malicious behaviors of malware.

- Comparison of adaptive performance between FedHGCDroid and other state-of-the-art studies on FL-based malware classification schemes

**Figure 15:**
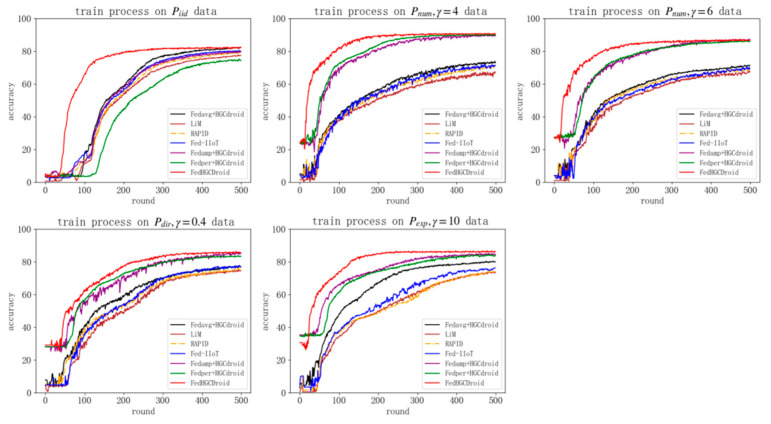
Performance of the FedHGCDroid and their FL-based malware classification schemes under different non-IID data settings.

As shown in the tables and Figure 15, FedHGCDroid achieves the highest classification accuracy in malware detection and classification tasks at different non-IID levels. At the same time, the FedHGCDroid’s convergence is the fastest. It shows that FedAdapt, the adaptive mechanism in FedHGCDroid, achieves the best adaptive performance in different non-IID scenarios. This is because the three existing FL-based malware classification models all adopt a single common model, or only carry out regularization constraints, and do not adapt to the distribution of malware on different clients. As a result, their adaptability to different non-IID scenarios is poor, and their accuracy is significantly decreased (about 6% on average). 

In the comparison experiment of supplementary personalization algorithm, [Fedamp + HGCdroid] (aggregations of similar data groups) and [Fedper + HGCdroid] (Fine-tune the top of the model) also adopt local model adjustment, so they can still maintain high accuracy in more non-IID scenarios, and even have some improvement compared with IID scenarios. It should be pointed out that this improvement in accuracy is due to the fact that the local personalization model is more suitable for the local data distribution characteristics (in non-IID scenarios, the local distribution may have fewer categories than the global distribution), thus slightly improving the overall accuracy. Compared with FedAmp and Fedper (state-of-the-art studies on personalized FL algorithms), proposed FedAdapt achieved approximately 2% higher average accuracy across different tasks and non-IID distributions. This is because the proposed FedAdapt adapts the characteristics of local distribution and global distribution more comprehensively in the local training stage (using meta-learning), global aggregation stage (using attention mechanism) and local fine-tuning stage (using meta-learning), respectively. Therefore, the proposed FedAdapt achieves higher adaptability than FedAmp and Fedper. 

It should be pointed out that the proposed FedAdapt is improved based on Fedavg (FL aggregation algorithm). Moreover, in three state-of-the-art studies of FL-based malware classification frameworks (Fed-IIoT [23], RAPID [22], LiM [21]), Fedavg is also used as the basic model aggregation function. 

Therefore, we further compare the proposed FedAdapt mechanism with Fedavg to verify the adaptive performance. Therefore, we further designed a comparison experiment between FedHGCDroid and [Fedavg+HGCdroid]. Furthermore, we test the influence of the same distribution type on the malware classification task at different γ levels (which will lead to different degrees of non-IID). The $P_{dir}^{\gamma }$ partition method was used to conduct the experiment, the data was scattered among five clients, and FedHGCDroid was used to conduct the experiment with the malware family classification as the target. By setting different parameter values, the data partitioning results of the same distribution type in different degrees of non-IID can be obtained.

**Figure 16:**
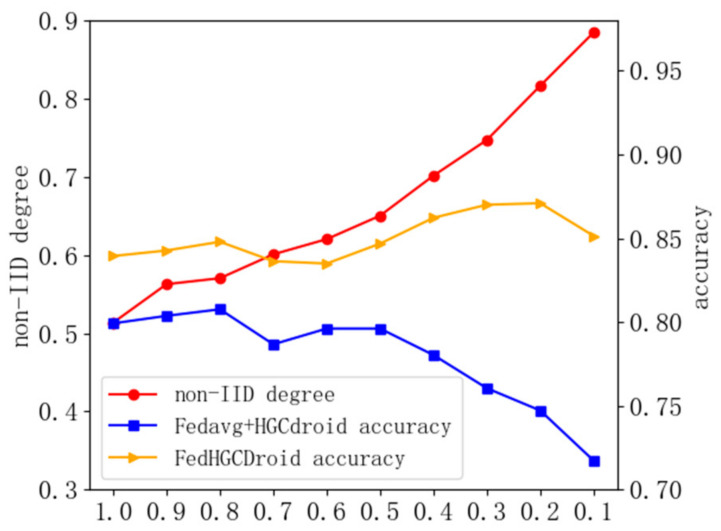
The influence of distributed difference value on Federated learning algorithm.

As shown in Figure 16, the abscissa represents the γ parameter. The ordinate represents the degree of non-IID of the current scene and the accuracy of the classification of the model (the task of malware family classification). Different parameters are set to obtain different degrees of non-IID partition results, and the classification accuracy of the model is obtained through experiments. The experimental results show that the classification accuracy of [Fedavg + HGCdroid] decreases with the increase in non-IID degree. However, the accuracy of the FedHGCDroid model is basically stable and improved to a certain extent, which further indicates the superiority of FedHGCDroid (FedAdapt) in adaptive performance, so that it can better handle the scenarios with non-IID distribution on the client.

## 5. Conclusions

In this article, we proposed an adaptive multi-Dimensional FL-based malware classification framework named FedHGCDroid for detecting and classifying malware in a privacy-preserving, highly accurate and adaptive manner. 

Firstly, we introduced an FL framework that enables distributed Android clients to collaboratively train a comprehensive Android malware classification model without transferring the user’s private data. The validity of the proposed framework is proved by compliance analysis in the data protection principles of GDPR. The analysis shows that the proposed framework basically solves the problem that users’ privacy is violated in the existing malware classification framework.

Secondly, we use a CNN and GNN to design a novel multi-dimensional malware classification model HGCDroid (as a local classification model in the proposed FL framework FedHGCDroid). The model encodes the API with multiple attributes and then uses the CNN network to capture the statistical features of malware and the GNN network to capture the graphical features of malware to obtain effective behavioral features of Android malware and then classify it accurately. Experimental results on the Androzoo dataset show that HGCDroid can achieve up to 91.3% accuracy and 91.25% F1-score in malware detection tasks, higher than the baseline model. It solved the problem that the existing framework was not comprehensive and effective enough to mine the behaviors of malware, thus improving the overall classification accuracy. To the best of our knowledge, this is the first attempt to combine statistical features with multi-attribute graph features for malware classification.

Finally, we innovatively combine the idea of meta-learning and attentional mechanisms and propose a contribution degree-based adaptive classifier training mechanism, namely FedAdapt. It adapts the characteristics of local distribution and global distribution more comprehensively in the local training stage (using meta-learning), global aggregation stage (using attention mechanism) and local fine-tuning stage (using meta-learning), respectively, to improve the adaptive performance of the proposed framework in scenarios with non-IID distributions on different clients. Experimental results show that FedAdapt can maintain the highest accuracy in malware detection and classification tasks under different degrees of non-IID settings, which is superior to the state-of-the-art models, proving its best adaptive performance. To the best of our knowledge, this is the first attempt to combine attentional mechanisms with meta-learning for adaptive performance optimization of the FL-based malware classification framework.

The research in this paper is helpful to motivate users or organizations to share malware data because it alleviates users’ concerns about their privacy. In this way, a wider range of malware data and computing resources from different organizations or users can be obtained to train a more efficient malware classification model. In addition, this paper enriches the research on the classification performance and adaptive performance of FL-based malware classification framework, which promotes the transformation process from the traditional centralized ML framework to the FL framework in the field of malware classification.

There are still some limitations to our proposed scheme that need to be worked out. In future work, under the proposed framework, we will study the detection methods of unknown malware families and the efficient online update mechanism on the client-side. In addition, this work also plans to further study a lightweight differential privacy approach for malware classifier training.

## Figures and Tables

**Table 1:** Summarization of previous related review articles in detecting malware (a).

Ref	Model	Features	Training Method	Statistical Feature	Graphical Feature	Privacy -Preserving	Adaptive
[12]	SVM	Permissions, Sensitive APIs	Centralized	√	-	-	-
[13]	N-gram	Operation sequences	Centralized	√	-	-	-
[9]	CNN	Method calls, App resources, Inter-Component Communication	Centralized	√	-	-	-
[11]	LSTM	Opcode	Centralized	√	-	-	-
[18]	DNN	Function call graph	Centralized	-	√	-	-
[15]	CNN	Control flow diagram	Centralized	-	√	-	-
[14]	Rotation Forest	API calls, Permission, System events	Centralized	√		-	-
[19]	GNN	API call graph	Centralized	-	√	-	-
[20]	CNN, AC-GAN	Image transformations, Bytes, Call graph	FL	√	-	√	-
[21]	DNN	Permissions, APIs, Intents	FL	√	-	√	-
[22]	CNN	Image transformations	FL	√	-	√	-
[23]	CNN	Permissions, Intents, and API calls	FL	√	-	√	-
Proposed FedHGCDroid	GNN, CNN	Function call graph, Permissions, API, Intent	FL	√	√	√	√

**Table 2:** Summarization of previous related review articles in detecting malware (b).

Ref	Key Contributions	Limitations
[12]	A lightweight method that detects malware on smartphones	These schemes only mine the characteristics of malware from a single dimension, and it is difficult to capture the essential behavior of malware comprehensively and effectively. Privacy issues during training are not considered.
[9]	Presented a novel classification approach based on dynamic analysis, which is robust to the obfuscation	
[11]	Propose a novel and efficient approach which uses LSTM to obtain the feature representations of opcode sequences of malware	
[19]	Feature mining of function call graph is carried out by graph embedding technique	
[15]	Propose an approach that transforms the control flow diagram into RGB images for the convolutional neural network for malware detection	
[14]	Propose a highly efficient method to extract API calls, permission rate, surveillance system events, and permissions as features	
[21]	Proposed a semi-supervised federated learning algorithm that works without user supervision	These schemes’ lack of adaptability to the problems that the non-IID distribution of malware on different clients
[22]	Introduces a performance-aware FL framework to reduce the communication overhead of device-level computing	
[23]	Proposed a robust FL-based framework, namely, Fed-IIoT, for detecting Android malware in the Internet of Things	

**Table 3:** List of abbreviations and mathematical symbols used.

Abbreviation and Symbol	Explanation
non-IID	Non-independent and identically distributed
FL	Federated learning
API	Application Programming Interface
DL	Deep learning
ML	Machine learning
GNN	Graph neural network
SVM	Support Vector Machine
CNN	Convolutional neural network
$D_{k}$	Local dataset
$D_{k}$	Size of local dataset
w	Global mel
$F\left(w\right)$	Global loss nction
$F_{k}\left(w\right)$	Local loss nction
X	Feature space of Android malware
Y	Category space of Android malware
x	Feature of Android malware
y	Category of Android malware
${\hat{w}}_{\;}{}^{\;}$	Initial model parameters
${\hat{w}}_{k}$	Gradient of initialization parameters
E	Mathematical expectation
θ	Coding vector of statistical features
G	Coding function call graph
V	The node set of function call graph
E	Edge set of function call graph
$h_{v}^{k}$	The embedded representation of node v in the function call graph at the k layer.
$N\left(s\right)$	Neighbor node sampling
τ	Represent the degree of non-IID
$\nabla L\left(w_{k}^{t}\right)$	Derivative of the loss function of the model to the weight of the model
GDPR	General Data Protection Regulation
γ	The scale parameter of the data partitioning method

**Table 4:** Neural network structure and parameters of CNN module.

Layer		Input × Output	Conv Kernel Size	Stride
CNN-1	Conv1D	1 × 64	3	1
	BatchNorm1D	64 × 64	-	-
	Relu	64 × 64	-	-
CNN-2	MaxPool1D	64 × 64	3	3
	Conv1D	64 × 128	3	1
	BatchNorm1D	128 × 128	-	-
	Relu	128 × 128	-	-
CNN-3	MaxPool1D	128 × 128	3	3
	Conv1D	128 × 256	5	1
	BatchNorm1D	256 × 256	-	-
	Relu	256 × 256	-	-
CNN-4	MaxPool1D	256 × 256	3	3
	Conv1D	256 × 256	5	1
	BatchNorm1D	256 × 256	-	-
	Relu	256 × 256	-	-
Pool	MaxPool1D	256 × 256	3	3

**Table 5:** Network structure and parameters of GNN module.

Layer		Input × Output
GNN-1	GraphConv	102 × 256
	Relu	256 × 256
GNN-2	GraphConv	256 × 256
	Relu	256 × 256
GNN-3	GraphConv	256 × 256
	Relu	256 × 256
Pool	Readout	1536

**Table 6:** Network structure and parameters of fully connected modules.

Layer		Input × Output	Layer		Input × Output
FC-1	Linear	2560 × 2560	FC-4	Linear	1536 × 1024
	LayerNorm	2560 × 2560		LayerNorm	1024 × 1024
	LeakyRelu	2560 × 2560		LeakyRelu	1024 × 1024
FC-2	Linear	2560 × 2560	FC-5	Linear	1024 × 1024
	LayerNorm	2560 × 2560		LayerNorm	1024 × 1024
	LeakyRelu	2560 × 2560		LeakyRelu	1024 × 1024
FC-3	Linear	2560 × Output	FC-6	Linear	1024 × 1024
FC-7	Sum	Output × Output			
SF	Softmax	Output × Output			

**Table 7:** Description of experimental dataset.

Task Type	Category	Quantity	Description
Malware detection	Benign	29,977	Normal application.
	Malicious	28,855	An application that performs malicious operations that cause losses to the user.
Malware type classification	Adware	5000	Flood a user’s device with unwanted ads, enticing them to click incorrectly.
	Trojan	3338	Masquerading software, damaging user devices, collecting sensitive data, deleting important files, and monitoring user activity.
	Riskware	5000	Collect users’ bank account information and payment records.
	Ransom	4322	Software that prevents the user from behaving normally and requires the user to pay a ransom to release control.
	Exploit	1225	Exploit system vulnerabilities to gain permissions by breaking the application sandbox.
	Spyware	2476	Transfer of personal information and data to places other than the Android device without the user’s consent.
	Downloader	4023	Remote download malicious code, frequently download and install operations.
	Fraudware	3776	To charge users in a deliberately deceptive manner.
Malware family classification	Adwo	1000	Display intrusive ads and gain privacy from the device.
	Airpush	1000	Trojan, take the initiative to push advertising to equipment notification bar.
	Dowgin	1000	Advertising module, collect device location, network, telephone sensitive information.
	Droidkunfu	1000	Trojans, which exploit vulnerabilities to send confidential information to remote servers.
	Jiagu	1000	Risk software, malicious packaging program.
	Kuguo	1000	Advertising module, steal sensitive information.
	Leadbolt	1000	Changes browser Settings to display ads in the notification bar.
	Revmob	1000	Get geolocation, download hidden executables.
	Wapsx	1000	Delivers AD content and displays unwanted ads in the notification bar.
	Youmi	1000	Steals user privacy, including location, phone, phone id, etc.

**Table 8:** Description of experimental environment.

	Component	Parameter
Hardware	CPU/GPU	Intel Golden 6240/NVIDIA A100 GPU
	Memory/Hard disk	64G/2T
Software	OS	CentOS 7.6, Cuda10.1
	Programming language	Python3.8
	Software tools	Vscode, Slrum
	Machine learning library	Pytorch1.8.1, Sklearn1.0.2, PyG2.0.1
	Other libraries	Androguard3.3.5, Numpy1.20.3, Matplotlib3.4.2, Gensim4.1.2, Conda4.8.2, Networkx2.6.3,

**Table 9:** Performance Metrics.

Performance Metrics	Calculation Formula
Accuracy	$\mathrm{ACC}=\frac{\mathrm{TP}+\mathrm{TN}}{\mathrm{TP}+\mathrm{TN}+\mathrm{FP}+\mathrm{FN}}$
Precision	$\mathrm{PRE}=\frac{\mathrm{TP}}{\mathrm{TP}+\mathrm{FP}}$
Recall	$\mathrm{REC}=\frac{\mathrm{TP}}{\mathrm{TP}+\mathrm{FN}}$
F1 score	$F1=\frac{2*\mathrm{PRE}*\mathrm{REC}}{\mathrm{PRE}+\mathrm{REC}}$

**Table 10:** Validation of HGCDroid model.

Experiment Task	Model	Accuracy	Precision	Recall	F1-Score
Malware detection	SVM (Arp et al. 2014) [12]	89.2	89.27	89.04	89.16
	DNN (Jiang et al. 2018) [18]	90.67	**91.3**	90.99	91.14
	CNN (Taheri et al. 2021) [23]	88.16	87.95	88.28	87.99
	GNN (Pektas et al. 2020) [19]	90.55	90.21	91.06	90.63
	HGCDroid	**91.3**	90.8	**92.79**	**91.29**
Malware type classification	SVM (Arp et al. 2014) [12]	72.08	72.68	73.62	73.14
	DNN (Jiang et al. 2018) [18]	78.14	76.72	77.69	77.38
	CNN (Taheri et al. 2021) [23]	79.13	79.71	78.61	79.07
	GNN (Pektas et al. 2020) [19]	80.5	82.66	81.59	82.12
	HGCDroid	**83.29**	**83.45**	**83.85**	**83.67**

**Table 11:** Compare the results of malware detection tasks.

Algorithm	$P_{iid}\;$	$P_{dir}^{\gamma }\;\;\gamma =0.2$	$P_{dir}^{\gamma }\;\;\gamma =0.6$	$P_{exp}^{\gamma }\;\;\gamma =0.2$	$P_{dir}^{\gamma }\;\;\gamma =15$
Fedavg + HGCdroid	91.37	90.12	89.77	90.06	91.91
LiM	88.58	81.02	85.11	86.71	78.57
RAPID	89.8	88.05	87.16	88.71	88.25
Fed-IIoT	90.51	87.47	88.62	88.96	80.54
Fedamp + HGCdroid	91.80	92.46	93.28	90.57	93.43
Fedper + HGCdroid	90.82	91.78	93.26	90.53	**93.53**
FedHGCdroid	**91.82**	**92.79**	**93.95**	**91.18**	93.19

**Table 12:** Comparison of malware type classification results.

Algorithm	$P_{iid}\;$	$P_{num}^{\gamma }\;\;\gamma =4\;$	$P_{num}^{\gamma }\;\;\gamma =6\;$	$P_{dir}^{\gamma }\;\;\gamma =0.4\;$	$P_{exp}^{\gamma }\;\;\gamma =10\;$
Fedavg + HGCdroid	**8** **2.** **16**	76.13	77.03	80.65	82.60
LiM	79.82	72.58	73.46	76.67	77.63
RAPID	80.04	77.33	76.94	78.5	77.48
Fed-IIoT	80.92	76.79	76.27	78.73	78.44
Fedamp + HGCdroid	81.47	83.87	85.41	83.75	83.19
Fedper + HGCdroid	78.25	82.86	83.79	81.99	83.11
FEDHGCdroid	81.79	**84.77**	**85.98**	**84.09**	**84.83**

**Table 13:** Comparison of malware family classification results.

Algorithm	$P_{iid}\;$	$P_{num}^{\gamma }\;\;\gamma =4\;$	$P_{num}^{\gamma }\;\;\gamma =6\;$	$P_{dir}^{\gamma }\;\;\gamma =0.4\;$	$P_{exp}^{\gamma }\;\;\gamma =10\;$
Fedavg + HGCdroid	81.01	76.77	78.63	76.98	74.67
LiM	76.63	73.15	72.34	75.82	72.96
RAPID	79.65	74.95	75.95	75.29	75.11
Fed-IIoT	79.93	74.63	77.67	78.42	74.86
Fedamp + HGCdroid	80.55	83.21	84.92	**86.48**	85.02
Fedper + HGCdroid	76.65	82.45	83.75	83.82	84.02
FedHGCdroid	**82.25**	**85.74**	**86.36**	86.27	**87.95**

## Data Availability

Not applicable.
